# Seeking Out High Risk Population: The Prevalence Characteristics and Outcome of Diabetic Patients of Arab Ethnicity Hospitalized in Internal Medical and Acute Coronary Units in Israel

**DOI:** 10.1155/2013/371608

**Published:** 2013-06-18

**Authors:** William Nseir, Shehadeh Haj, Basma Beshara, Julnar Mograbi, Ohad Cohen

**Affiliations:** ^1^The Holy Family Hospital, Nazareth, Israel; ^2^The Medical Department, Nazareth Hospital, EMMS, Nazareth, Israel; ^3^Sackler School of Medicine, Tel Aviv University, Israel

## Abstract

*Aims*. To seek high risk population for diabetes and to improve their health care by investigating the characteristics and outcome of hospitalization in hospitals with predominant Arab patients in Northern Israel. *Methods*. Retrospective analysis of the prevalence of diabetes and the outcome of diabetic in comparison to nondiabetic patients hospitalized in the internal medicine and intensive cardiac units in two major hospitals with one-year postdischarge data between 1.1.2009 and 31.12.2009. *Results*. Thirty-nine percent of the patients were diagnosed with diabetes. The preponderance of women in the diabetes group was noted. Diabetic patients had an increase in the duration of hospitalization (*P* = 0.0008), with one hospital having a high readmission rate for the diabetic patients. The average glycemia during hospitalization exceeded the recommended threshold of 180 mg% without major changes in the therapeutic regimens in comparison to preadmission regimens. *Conclusions*. Arab populations, women in particular, in westernizing societies are at high risk for diabetes which exemplifies as high rate of patients with diabetes among hospitalized patients. Areas for intervention during hospitalization and at predischarge have been identified to improve health outcomes and prevent readmissions.

## 1. Introduction

The prevalence of diabetes is increasing globally, associated with an increase in obesity and in sedentary lifestyle [1–3]. This is associated with morbidity and mortality due to the effects of hyperglycemia related complication and by its association with atherosclerosis-heart disease and stroke, as the leading causes for mortality in diabetics. Hospitalization for acute internal and coronary disorders is therefore high among patients with diabetes; estimates from several studies show that the percentage of patients with diabetes admitted is up to three times the percent of diabetes in the relevant population [4]. 

 The increase in the prevalence diabetes and obesity is, however, not distributed evenly between different racial and ethnic groups, with some groups showing higher susceptibility to environmental changes brought by immigration or rapid socioeconomic changes. 

The Arab population in Israel has recently been observed to have an alarming high prevalence of diabetes, reaching 50% in women above 50 years old [5]. This high prevalence seems to be higher than observed in Arab countries or in Arab immigrants to the USA or Western Europe. The unique situation of this population calls for both studies and intervention on a national level to promote health and prevent the consequences of the metabolic perturbation [6].

Considering the high prevalence of diabetes in the Arab population in Israel, we hypothesized that we will find substantial number of patients in the internal and the intensive coronary unit (ICU) wards diagnosed and undiagnosed diabetic patients. Seeking such a high risk population will enable the characterization of demographic, clinical state and diabetes related parameters. This data will serve to form intervention strategy in order to enhance health related outcomes.

## 2. Material and Methods

### 2.1. Population

The study was performed at the Internal Medicine wards and ICU of the Holy Family (HFH) Hospital and the Nazareth Hospital in Nazareth. The HFH hospital (established 1882) has 32 beds in the Internal Ward and ICU. The Nazareth Hospital was established by the Edinburgh Medical Mission Society in 1861 in which 40 beds are in the internal ward and the ICU. Both wards have affiliated with the Bar Ilan medical school, Zefat. These two hospitals serve the population of Nazareth and surrounding villages and towns. Estimated population served is 463,000. The majority of the population is urban. The rural population has shifted from mainly an agrarian society toward an urban like society. Both hospitals serve mainly the Arab population in Northern Israel with an estimated population of 253,000.

### 2.2. Definitions

 Diabetes was defined as either known or unknown diabetes. 

 Known Diabetes: pervious diagnosis and/or treatment with diabetes related medication. 

Unknown Diabetes: two measurements of blood glucose > 200 mg% and/or HbA1c > 6.5%.

Dwelling was defined as urban residing in the areas of major cities Nazareth and Acre; rural-residing in the areas of smaller towns and villages. 

### 2.3. Data Retrieved

Data on all patients hospitalized in the Internal Medicine and ICU units in HFH and Nazareth hospitals between 1.1.2009 and 31.12.2009 were reviewed. Charts of all diabetics were probed for data concerning demographics and previous diagnosis, and data pertaining to the hospitalization cause and the outcomes. Population census was searched for one-year after discharge for mortality data. All patients who were hospitalized during the months: January, April, July, and October served as a control group. Data was collected similarly to the diabetes cohort.

### 2.4. Statistical Methods

All measured variables and derived parameters were tabulated by descriptive statistics. For descriptive statistics summary tables were provided giving sample size, absolute and relative frequency for categorical variables and sample size, arithmetic mean, standard deviation, median, minimum, and maximum for continuous variables.

The following statistical tests were used in the analysis of the data presented in this study.

 
*Chi-square test* was applied for testing the statistical significance of the differences in frequency of categorical variables between the study groups.
 
*The two-sample t-test* was applied for testing the statistical significance of the differences of continuous variables between the study groups.
 
*Analysis of Covariance (ANOVA)* was applied for comparing the differences of continuous variables between the outcomes of patients.
 
*Logistic Regression* was applied using patient's death as outcome and glucose on day 1 as predictor variable.
 
*Multiple Regression* was applied using days of hospitalization as outcome and glucose on day 1 as predictor variable.

All tests applied were two-tailed, and *P* value of 5% or less was considered statistically significant. The data was analyzed using the SAS version 9.1 (SAS Institute, Cary, NC).

## 3. Results

Population: there were 1489 diabetic patients admitted during the study year. The number of all patients hospitalized one or more during the study year was 3784. Thus, the proportion of diabetic patients hospitalized for one or more times during the study year was 39%. 

Patient demographics show the preponderance of the Arab patients admitted: 92.8% of diabetic and 90.7% in the control group (*P* = nonsignificant (NS)). The minority were Jews (3.45, 5.1%, resp.) or other ethnic groups (*P* = NS). There was a significant difference between groups regarding patients domicile. More patients with diabetes reported residence in rural versus urban areas than in the control group (Table 1). 

Clinical characteristics at admission differed between patients with diabetes and controls (Table 2). Diabetic patients admitted were significantly older than control 66.53 ± 12.72 years and 54.26 ± 21.53 years, respectively (*P* < 0.0001). Gender was significantly different between the control and the diabetic groups. Of the diabetic patients admitted, 52.9% were women in comparison to 45.0% of the control group (*P* = 0.0003). 

Weight did not differ between groups 82.39 ± 49.12 versus 85.08 ± 19.08 (*P* = 0.47). There were significantly less smokers among the diabetic patients (Table 2).

**Figure 1:**
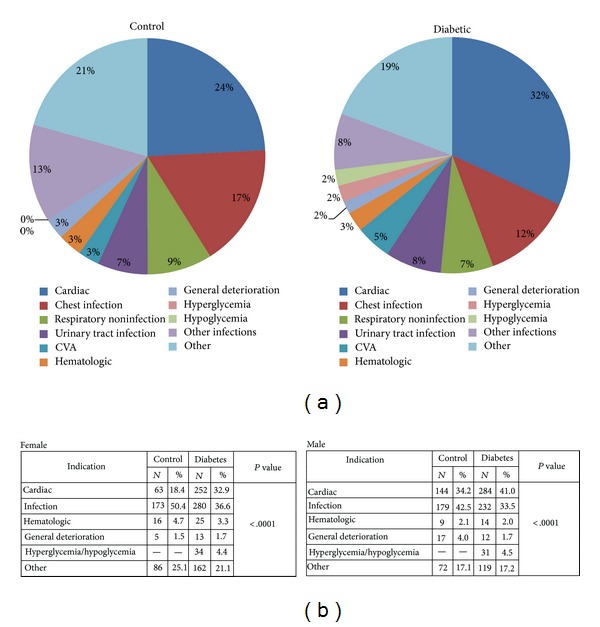
Analysis between controls to diabetes patients (2 hospitals). Summary of admission indications in percentage for the nondiabetic and diabetic groups (a) and according to gender by the diabetic and nondiabetic groups (b).

The indications for hospitalization were different between diabetes and controls and between genders: the main difference was in the higher occurrence of hospitalization due to atherosclerotic related diseases—both cardiovascular disease and strokes in the diabetic group versus controls (37% versus 27%, resp., *P* < 0.001) (Figure 1). Significant Differences in the indications for hospitalization were maintained within the gender in each group. While only 18.4% of control women were hospitalized for cardiac indications, 32.9% of diabetic women were hospitalized for cardiac reasons—a similar rate as in the nondiabetic men (34.2%). In comorbidities, we also detected significant difference between groups with a noticeable higher renal disease in the diabetic group (16.6% versus 7.9%) (Table 3).

### 3.1. Outcome Results

Hospitalization: duration of hospitalization was significantly longer for the diabetic patients than for control patients: 3.71 ± 2.99 days versus 3.27 ± 2.97 days, respectively (*P* = 0.0008) (Table 2). No significant differences in discharge status were noted between the two groups with the majority of patients being home discharged and with a low rate of in-hospital death—2.6% control and 2.3% diabetic (Table 4). One-year outcome was poorer in the diabetic group but without reaching statistical significance with a major difference in readmission rates in the diabetic group between the two hospitals. Mortality during the first year following discharge was 11.5% in the control versus 13.2% in the diabetic group (*P* = 0.48). A high readmission rate for both groups 19.9% and 20.3% control and diabetic group respectively, but there was an inequality between the two hospitals with one hospital demonstrating a significant difference between the two groups in the rate of readmission being significantly higher in the diabetic group during the first year after discharge in comparison to the control readmission rate. 

### 3.2. Intrahospital Glycemic Control

**Figure 2:**
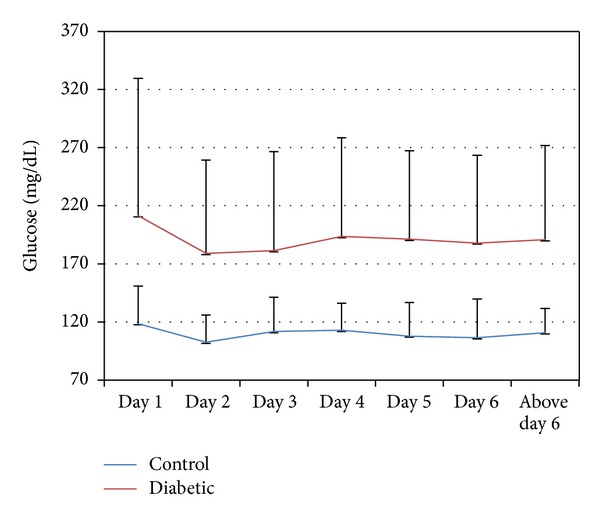
Analysis between Controls to Diabetes patients (2 hospitals). Blood glucose levels by hospitalization day.

Average daily glucose levels during hospitalization as described in Figure 2 demonstrate a decline in values from day one 211.4 (±118.1) mg/dL to less than 200 mg/dL and thereafter stable levels while glucose levels were stable in the control group (Figure 2). The glucose level at admission was a significant and an independent predictor for longer hospitalization and death in the nondiabetic but not in the diabetic group (data not shown). 

### 3.3. Indication for Hospitalization

As a group, indications for hospitalization differed between patients with diabetes and controls. For both groups, the leading indication for hospitalization was cardiovascular disease, but cardiovascular disease and urinary tract infection were more prevalent as an indication for hospitalization in the diabetic group (36.8% versus 27.1% and 7.7% versus 6.9%, resp.) while chest infections were significantly less prevalent in the diabetic group versus control group (12.5% versus 16.9%, resp.).

### 3.4. Diabetes Related Treatment Modifications

 The glucose lowering treatment for the patients with diabetes, prior to their hospitalization, was diet alone in 13.1%, oral therapy in 60%, insulin in 19%, and combination therapy in 7% of the patients. At discharge, there was no significant shift in the treatment paradigm in the total group (Table 5). 

## 4. Discussion

 This study sheds light on the characteristics of hospitalized patients with diabetes in hospitals serving a high risk for diabetes population. We found high percentage of diabetes among the patients hospitalized in the internal medicine wards and cardiac intensive units, reflecting the high prevalence of diabetes in the population. 39.3% of total patient admitted to the internal medical ward and intensive care unit were diabetic. There was a female preponderance among patients admitted with diabetes 52.9% while only 45.0% of patients without diabetes, hospitalized, were women (*P* = 0.0003). All this reflects the alarming prevalence of obesity and diabetes among adult Arab women in northern Israel. It has been recently reported that the prevalence of overweigh and obesity among Arab woman over 40 years of age who attend primary health clinic is 74% [7–9] with diabetes near 50% [5]. This novel observation is seminal by bringing attention to this specific group of women with high morbidity risk and now observing high rate of hospitalization. Interestingly, diabetic women tend to have similar prevalence of coronary heart disease as main indication for admission, as for nondiabetic men (32.9% versus 34.2%, resp.). 

 As cardiovascular disease and urinary tract infection were more prevalent as an indication for hospitalization in the diabetic group, prevention of cardiovascular disease and urinary tract infection should therefore be reinforced particularly in the group of Arab diabetic women within diabetic population. 

 Patients with diabetes had a significant greater length of stay than hospitalized patients without diabetes, similar to data described in previous publications [10–12]. The reasons might be multifactorial, and it is important to note that interventions with chronic disease management programs for outpatients [13] and diabetes team consultation during hospitalization [10] have been proved to be an effective means to reduce the likelihood and duration of hospitalizations for individuals with diabetes. Implementing similar strategies in this high risk group and evaluating the impact of this intervention on outcomes of rate and recurrence of hospitalization and hospital length of stay is therefore of paramount importance. In this study, we can additionally identify areas for intervention; as is evident in Table 5, the stay in the hospital did not affect the patients treatment regimens, although glycemic levels at discharge were above the target (mean glucose levels at discharge >190 mg/dL). Furthermore, in only a minority of patients, an HbA1c measurement was performed during hospitalization. These issues reflect the current practice and constraint on hospitalization length and on the performance of blood tests, pushing toward shorter hospital stay and cutting back on performing blood tests not directly relevant to the immediate hospital care. It will be of interest to examine whether interventions as described above [13] will cause changes in treatment regimens with improvement in long-term outcomes and readmissions. 

 The glycemic control goals in nonsurgical hospitalized patients continue to be debated, but a consensus defining upper limits below 180 m/dL in noncritical patients is well based on outcomes studies and is therefore recommended by recent position papers [14]. Average glucose levels during hospitalization were above 180 mg% throughout the hospital stay (Figure 2); therefore, as noted there is a need for team consultation during hospitalization tailored to hospitals that provide medical care to populations in high risk for diabetes. 

Results of this study show the impact of the high prevalence of diabetes on the hospitalization of Arab patients in Northern Israel, with specific emphasis on the burden of diabetes of the Arab women. It calls attention to the need of in-hospital consultation team and postdischarge plan. Information from the cohort described within this study might be applicable to other medical centers providing large population of Arab descent [15–17].

## Supplementary Material

Summary of Glucose by hospitalization day, Creatine and Frequency of Indications are presented in the Supplement.

## Figures and Tables

**Table 1:** Analysis between controls and diabetes patients (2 hospitals), demographic data.

	Control		Diabetes		*P* value
	*N*	%	*N*	%	
Gender					
Male	421	55.0	701	47.1	0.0003
Female	344	45.0	788	52.9	
Ethnicity					
Arab	710	92.8	1351	90.7	0.1551
Jew	26	3.4	76	5.1	
Other	29	3.8	63	4.2	
Dwelling					
Urban	447	58.4	364	24.4	<0.0001
Non urban	317	41.4	1121	75.2	
UK	1	0.1	5	0.3	
Smoker					
No	374	48.9	930	65.9	<0.0001
Yes	244	31.9	372	26.3	
UK	147	19.2	110	7.8	

**Table 2:** Analysis between controls and diabetes patients (2 hospitals); summary of data at admission.

Baseline data	Control						Diabetes						*P* value
	*N*	Mean	Std.	Median	Min.	Max.	*N*	Mean	Std.	Median	Min.	Max.	
Age at hospitalization (year)	758	54.26	21.53	53.79	14.01	100.3	1392	66.53	12.72	67.21	16.18	98.90	<0.0001
Weight	179	82.39	49.12	76.00	42.00	676.0	795	85.08	19.08	83.00	45.00	169.0	0.4722
Height	170	164.5	12.79	164.0	95.00	193.0	670	167.5	101.5	162.0	54.00	2007	0.4545
HbA1c							106	8.84	2.51	8.15	5.10	17.70	
Duration of diabetes							821	10.80	5.89	10.00	0.00	48.00	
Days of hospitalization	761	3.27	2.97	3.00	0.00	29.00	1486	3.71	2.99	3.00	0.00	30.00	0.0008

**Table 3:** Analysis between controls and Diabetes patients (2 hospitals), frequency of comorbidity.

Comorbidity	Control		Diabetes		*P* value
	*N*	%	*N*	%	
Carcinoma	31	6.8	62	4.9	<0.0001
Neurological	34	7.5	64	5.0	
Psychiatric	19	4.2	12	0.9	
Renal	36	7.9	211	16.6	
Respiratory	72	15.9	119	9.4	
Cardiac	135	29.7	397	31.3	
Hypertension	127	28.0	404	31.8	

**Table 4:** Analysis between controls and diabetes patients (2 hospitals), frequency of outcome.

	Control		Diabetes		*P* value
	*N*	%	*N*	%	
Outcome					
Home discharge	727	95.0	1419	95.2	0.8734
Institutional discharge	18	2.4	37	2.5	
Death	20	2.6	34	2.3	
Outcome one year					
Alive	460	68.7	990	66.5	0.4805
Dead	77	11.5	197	13.2	
Readmission	133	19.9	302	20.3	

**Table 5:** Analysis between controls and diabetes patients (2 hospitals), frequency of diabetes treatments.

	*N*	%
Preadmission diabetes treatment		
None	178	13.1
Oral	823	60.6
Insulin	262	19.3
Combination	95	7.0
Discharge diabetes treatment		
None	216	16.1
Oral	755	56.3
Insulin	277	20.6
Combination	94	7.0
