# Supplementary material for: Seeking Out High Risk Population: The Prevalence Characteristics and Outcome of Diabetic Patients of Arab Ethnicity Hospitalized in Internal Medical and Acute Coronary Units in Israel
Source: Int J Endocrinol. 2013 Jun 18;2013:371608. doi: 10.1155/2013/371608 (PMC3703333; doi:10.1155/2013/371608)
Supplement: Supplementary file 1 — Summary of Glucose by hospitalization day, Creatine and Frequency of Indications are presented in the Supplement. [file 371608.f1.docx]

***Analysis between Controls to Diabetes patients (2 hospitals)***

***Table 1 Demographic data***

| **Gender** | **Control** | | | | **Diabetes** | | | | | **P value** |  |
| --- | --- | --- | --- | --- | --- | --- | --- | --- | --- | --- | --- |
|  | **N** | | **%** | | **N** | | | **%** | | 0.0003 |  |
| Male | 421 | | 55.0 | | 701 | | | 47.1 | |  |  |
| Female | 344 | | 45.0 | | 788 | | | 52.9 | |  |  |
| **Ethnicity** | | 710 | 92.8 | | | 1351 | | | 90.7 | 0.1551 |  |
| Arab | |  |  | | |  | | |  |  |  |
| Jew | | 26 | 3.4 | | | 76 | | | 5.1 |  |  |
| Other | | 29 | 3.8 | | | 63 | | | 4.2 |  |  |
| **Dwelling** | | 447 | 58.4 | | | 364 | | | 24.4 | <.0001 |  |
| Urban | |  |  | | |  | | |  |  |  |
| Non urban | | 317 | 41.4 | | | 1121 | | | 75.2 |  |  |
| UK | | 1 | 0.1 | | | 5 | | | 0.3 |  |  |
| **Smoker** |  | | | | | | | | |  | |
| No | 374 | | | 48.9 | | | 930 | 65.9 | | <.0001 | |
| Yes | 244 | | | 31.9 | | | 372 | 26.3 | |  | |
| UK | 147 | | | 19.2 | | | 110 | 7.8 | |  | |

***Table 2: Summary of Data At Admission***

| **Baseline Data** | **Control** | | | | | | **Diabetes** | | | | | | **P value** |
| --- | --- | --- | --- | --- | --- | --- | --- | --- | --- | --- | --- | --- | --- |
|  | **N** | **Mean** | **Std** | **Median** | **Min** | **Max** | **N** | **Mean** | **Std** | **Median** | **Min** | **Max** |  |
| **Age at Hospitalization (year)** | 758 | 54.26 | 21.53 | 53.79 | 14.01 | 100.3 | 1392 | 66.53 | 12.72 | 67.21 | 16.18 | 98.90 | <.0001 |
| **Weight** | 179 | 82.39 | 49.12 | 76.00 | 42.00 | 676.0 | 795 | 85.08 | 19.08 | 83.00 | 45.00 | 169.0 | 0.4722 |
| **Height** | 170 | 164.5 | 12.79 | 164.0 | 95.00 | 193.0 | 670 | 167.5 | 101.5 | 162.0 | 54.00 | 2007 | 0.4545 |
| **HbA1c** |  | | | | | | 106 | 8.84 | 2.51 | 8.15 | 5.10 | 17.70 |  |
| **Duration of diabetes** |  |  |  |  |  |  | 821 | 10.80 | 5.89 | 10.00 | 0.00 | 48.00 |  |
| **Days of Hospitalization** | 761 | 3.27 | 2.97 | 3.00 | 0.00 | 29.00 | 1486 | 3.71 | 2.99 | 3.00 | 0.00 | 30.00 | 0.0008 |

***Table 3: Frequency of Co-morbidity***

| **Co-morbidity** | **Control** | | **Diabetes** | | **P value** |
| --- | --- | --- | --- | --- | --- |
|  | **N** | **%** | **N** | **%** |  |
| Carcinoma | 31 | 6.8 | 62 | 4.9 | <.0001 |
| Neurological | 34 | 7.5 | 64 | 5.0 |  |
| Psychiatric | 19 | 4.2 | 12 | 0.9 |  |
| Renal | 36 | 7.9 | 211 | 16.6 |  |
| Respiratory | 72 | 15.9 | 119 | 9.4 |  |
| Cardiac | 135 | 29.7 | 397 | 31.3 |  |
| Hypertension | 127 | 28.0 | 404 | 31.8 |  |

***Figure 1: Summary of Admission Indications in percentage for the nondiabetic and diabetic groups(A)and according to gender by the diabetic and nondiabetic groups( B).***

1. ***Control***
2. ***Diabetic***

| 1. **Female** | | | | |  |
| --- | --- | --- | --- | --- | --- |
| **Indication** | **Control** | | **Diabetes** | | **P value** |
|  | **N** | **%** | **N** | **%** |  |
| Cardiac | 63 | 18.4 | 252 | 32.9 | <.0001 |
| Infection | 173 | 50.4 | 280 | 36.6 |  |
| Hematologic | 16 | 4.7 | 25 | 3.3 |  |
| General deterioration | 5 | 1.5 | 13 | 1.7 |  |
| Hyperglycemia/Hypoglycemia | . | . | 34 | 4.4 |  |
| Other | 86 | 25.1 | 162 | 21.1 |  |

| ***B.* Male** | | | | |  |
| --- | --- | --- | --- | --- | --- |
| **Indication** | **Control** | | **Diabetes** | | **P value** |
|  | **N** | **%** | **N** | **%** |  |
| Cardiac | 144 | 34.2 | 284 | 41.0 | <.0001 |
| Infection | 179 | 42.5 | 232 | 33.5 |  |
| Hematologic | 9 | 2.1 | 14 | 2.0 |  |
| General deterioration | 17 | 4.0 | 12 | 1.7 |  |
| Hyperglycemia/Hypoglycemia | . | . | 31 | 4.5 |  |
| Other | 72 | 17.1 | 119 | 17.2 |  |

| **Outcome** | **Control** | | **Diabetes** | | **P value** |
| --- | --- | --- | --- | --- | --- |
|  | **N** | **%** | **N** | **%** |  |
| Home discharge | 727 | 95.0 | 1419 | 95.2 | 0.8734 |
| Istitutional discharge | 18 | 2.4 | 37 | 2.5 |  |
| Death | 20 | 2.6 | 34 | 2.3 |  |

| **Outcome One Year** | **Control** | | **Diabetes** | | **P value** |
| --- | --- | --- | --- | --- | --- |
|  | **N** | **%** | **N** | **%** |  |
| **Alive** | 460 | 68.7 | 990 | 66.5 | 0.4805 |
| **Dead** | 77 | 11.5 | 197 | 13.2 |  |
| **Readmission** | 133 | 19.9 | 302 | 20.3 |  |

***Table 4: Frequency of Outcome***

*Figure 2: Blood glucose levels by hospitalization day.*

| **Preadmission diabetes treatment** | **N** | **%** |
| --- | --- | --- |
| None | 178 | 13.1 |
| Oral | 823 | 60.6 |
| Insulin | 262 | 19.3 |
| Combination | 95 | 7.0 |

| **Discharge diabetes treatment** | **N** | **%** |
| --- | --- | --- |
| None | 216 | 16.1 |
| Oral | 755 | 56.3 |
| Insulin | 277 | 20.6 |
| Combination | 94 | 7.0 |

***Table 5: Frequency of Diabetes treatments***

***SUPPLEMENT***

***Table S1: Summary of Glucose***

| **Glucose** | **Control** | | | | | | **Diabetes** | | | | | | **P value** |
| --- | --- | --- | --- | --- | --- | --- | --- | --- | --- | --- | --- | --- | --- |
|  | **N** | **Mean** | **Std** | **Median** | **Min** | **Max** | **N** | **Mean** | **Std** | **Median** | **Min** | **Max** |  |
| **Day 1** | 754 | 118.5 | 32.40 | 110.0 | 48.00 | 437.0 | 1469 | 211.4 | 118.1 | 185.0 | 16.00 | 1799 | <.0001 |
| **Day 2** | 560 | 102.5 | 23.51 | 97.00 | 17.00 | 245.0 | 1209 | 178.9 | 80.47 | 160.0 | 47.00 | 708.0 | <.0001 |
| **Day 3** | 124 | 111.8 | 29.31 | 105.0 | 76.00 | 278.0 | 615 | 181.3 | 85.41 | 161.0 | 32.00 | 1037 | <.0001 |
| **Day 4** | 48 | 112.8 | 23.22 | 108.0 | 80.00 | 180.0 | 322 | 193.5 | 84.88 | 177.0 | 10.00 | 500.0 | <.0001 |
| **Day 5** | 24 | 107.8 | 28.93 | 96.50 | 73.00 | 175.0 | 169 | 191.2 | 76.10 | 176.0 | 78.00 | 450.0 | <.0001 |
| **Day 6** | 14 | 106.4 | 33.28 | 95.00 | 73.00 | 197.0 | 94 | 187.9 | 75.53 | 180.0 | 77.00 | 409.0 | <.0001 |
| **Above 6** | 489 | 110.6 | 20.89 | 107.5 | 59.33 | 232.5 | 914 | 190.8 | 80.88 | 173.2 | 26.00 | 1011 | <.0001 |

***Table S2: Summary of Creatine***

| **Creatine** | **Control** | | | | | | **Diabetes** | | | | | | **P value** |
| --- | --- | --- | --- | --- | --- | --- | --- | --- | --- | --- | --- | --- | --- |
|  | **N** | **Mean** | **Std** | **Median** | **Min** | **Max** | **N** | **Mean** | **Std** | **Median** | **Min** | **Max** |  |
| **Admission creatine** | 749 | 1.00 | 0.95 | 0.80 | 0.18 | 12.60 | 1480 | 1.36 | 1.29 | 0.94 | 0.20 | 11.9 | <.0001 |
| **Discharge creatine** | 587 | 0.97 | 1.08 | 0.80 | 0.00 | 19.00 | 1210 | 1.28 | 1.20 | 0.90 | 0.06 | 11.20 | <.0001 |

***Table S3: Frequency of Indications***

| **Indication** | **Control** | | **Diabetes** | | **P value** |
| --- | --- | --- | --- | --- | --- |
|  | **N** | **%** | **N** | **%** |  |
| Cardiac | 185 | 24.2 | 465 | 31.9 | <.0001 |
| Chest infection | 129 | 16.9 | 182 | 12.5 |  |
| Respiratory noninfection | 68 | 8.9 | 105 | 7.2 |  |
| Urinary tract infection | 53 | 6.9 | 113 | 7.7 |  |
| CVA | 22 | 2.9 | 71 | 4.9 |  |
| Hematologic | 25 | 3.3 | 39 | 2.7 |  |
| General deterioration | 22 | 2.9 | 25 | 1.7 |  |
| Hyperglycemia | . | . | 33 | 2.3 |  |
| Hypoglycemia | . | . | 32 | 2.2 |  |
| Other infection | 102 | 13.4 | 112 | 7.7 |  |
| Other | 158 | 20.7 | 282 | 19.3 |  |
